# Association Between BDNF Gene Variant Rs6265 and the Severity of Depression in Antidepressant Treatment-Free Depressed Patients

**DOI:** 10.3389/fpsyt.2020.00038

**Published:** 2020-02-12

**Authors:** Innokentiy S. Losenkov, Nathaniël J. V. Mulder, Lyudmila A. Levchuk, Natalya M. Vyalova, Anton J. M. Loonen, Fokko J. Bosker, German G. Simutkin, Anastasiia S. Boiko, Nikolay A. Bokhan, Bob Wilffert, Eelko Hak, Amand F. Schmidt, Svetlana A. Ivanova

**Affiliations:** ^1^Mental Health Research Institute, Tomsk National Research Medical Center of the Russian Academy of Sciences, Tomsk, Russia; ^2^Unit of PharmacoTherapy, -Epidemiology & -Economics, Groningen Research Institute of Pharmacy, University of Groningen, Groningen, Netherlands; ^3^Policy Office for Quality and Innovation of Care (BZI), GGZ Westelijk Noord-Brabant, Halsteren, Netherlands; ^4^University Centre for Psychiatry, University Medical Centre Groningen, University of Groningen, Groningen, Netherlands; ^5^Department of Psychotherapy and Psychological Counseling, National Research Tomsk State University, Tomsk, Russia; ^6^Department of Psychiatry, Addictology and Psychotherapy, Siberian State Medical University, Tomsk, Russia; ^7^Department of Clinical Pharmacy and Pharmacology, University Medical Center Groningen, University of Groningen, Groningen, Netherlands; ^8^Division for Control and Diagnostics, School of Non-Destructive Testing & Security, National Research Tomsk Polytechnic University, Tomsk, Russia

**Keywords:** major depressive disorder, prolactin, brain derived neurotrophic factor, blood levels, genotypes, rs6265

## Abstract

**Background:**

Brain-derived neurotrophic factor (BDNF) plays an important role in neuronal plasticity, and its dysregulation has been associated with the pathogenesis of mood and anxiety disorders. Prolactin (PRL) is a pituitary hormone which is also produced as a cytokine by immune cells and could be a neurotrophic factor regulating the functional activity of stress-related mechanisms.

**Aim:**

To investigate the possible relationship between depressive state and BDNF and PRL genotypes or levels with special reference to severity of depression.

**Methods:**

Participants of 18–70 years with a clinical diagnosis of depressive disorder of at least moderate severity were included. These patients had not been treated with antidepressant drugs before admission to hospital during the preceding period of the last 6 months, and 54.5% had never been treated with antidepressant drugs during their entire life. The DNA was genotyped for rs1341239 within the prolactin and for rs6265, rs7124442, and rs11030104 within the BDNF gene. Rs11030104 violated the Hardy-Weinberg equilibrium distribution and was excluded from further analyses. BDNF and prolactin concentration was measured in serum by MAGPIX multiplex analyzer (Luminex, USA) using MILLIPLEX^®^ MAP kit (Merck, Germany). Genetic associations were determined by sequentially regressing prolactin, BDNF, 17-items Hamilton's Depression (HAMD-17) and Clinical Global Impression scale, Severity (CGI-S) ratings, and depression (absent/present) on the available SNPs. Genetic associations were evaluated assuming an additive model.

**Results:**

A total of 186 depressed patients (of which 169 were women) and 94 healthy controls (67 women) were genotyped. After excluding subjects without genetic information on all three study SNPs, 217 remained of whom 138 suffered from depression. Within depressed patients we observed an association of rs6265 with HAMD-17: mean difference (MD) 2.33 (95%CI 0.49; 4.16; *p* = 0.014) and CGI-S: MD 0.38 (95%CI 0.09; 0.66; *p* = 0.011). No significant association was observed between the prolactin SNP rs1341239 and prolactin levels. Similarly the mean differences of BDNF SNPs did not show an association with BDNF: rs6265 −0.042 ln(pg/ml) (95%CI −0.198; 0.113), and rs7124442 0.006 ln(pg/ml) (95%CI −0.117; 0.130). No other association reached statistical significance.

**Conclusion:**

We observed a significant association between BDNF gene variant rs6265 and the severity of depression in newly admitted, antidepressant treatment-free, depressed patients. Actual PRL and BDNF levels were not elevated sufficiently in depressed patients to reach statistical significance and were not associated with the studied genotypes.

## Introduction

Brain-derived neurotrophic factor (BDNF), discovered in 1982 (1), is a member of the neurotrophin family, also including nerve growth factor (NGF), neurotrophin-3 (NT-3), and neurotrophin-4 (NT-4) (2). Numerous genetic, pharmacological and behavioral studies have linked the dysregulation of BDNF to major psychiatric and neurological disorders, including mood and anxiety disorders (3–5).

The human *BDNF* gene, located on human chromosome 11p14.1, has a complex structure, and its expression is under sophisticated epigenetic control (3, 6). *BDNF* genotype and expression can be expected to modulate BDNF levels and/or its neuroplastic effects and may, therefore, affect the vulnerability to develop mood disorders. Most often studied is the single nucleotide polymorphism (SNP) with a G to A transition on position 196 in exon 5 of *BDNF* (rs6265), which results in an amino acid substitution (valine [Val] to methionine [Met]) at codon 66 in the precursor BDNF peptide sequence (BDNF Val66Met). The Met (A) allele induces impaired functioning of some processes involved in the regulation of extracellular levels of BDNF (7). However, in spite of these functional consequences, systematic reviews of the results of association studies suggest that this BDNF genotype does neither exert a major influence on the development of depression (7–9), nor on the serum BDNF levels (10). However, the influence of the Val66Met BDNF (rs6265) polymorphism may depend on age group, gender, and ethnicity (7, 11–14). Moreover, according to other meta-analyses interesting results were obtained concerning an association with the results of drug treatment (more specifically in Asian gender and when treated with selective serotonin reuptake inhibitors; SSRIs) (15, 16) and vulnerability with life stress (17).

The 199 amino-acid hormone prolactin (PRL) is synthesized and secreted in a pulsatile manner (~ 10 peaks per day in young adults) by lactotroph cells of the adenohypophysis (18). The gene that encodes prolactin (PRL) is on chromosome 6p21 (19, 20). In humans, the *PRL* locus consists of a single gene that contains five coding exons, which is controlled by a pituitary-specific promoter, and a non-coding exon, which is controlled by an alternative promoter. The latter promoter drives expression in non-pituitary tissues (21). Thus, apart from being a pituitary hormone, PRL has also a role as a cytokine produced by immune cells; its receptor belongs to the family of cytokine receptors type 1 (22). A functional polymorphism in the PRL gene, − 1149 G/T (rs1341239) has been identified of which the G allele was associated with increased extrapituitary promoter activity and increased levels of lymphocyte PRL mRNA (23). Hence, PRL could be one of the cytokines regulating the functional activity of stress-related mechanisms (5). Ivanova and colleagues have previously revealed the existence of a significant association between the polymorphic variant rs1341239 and the development of hyperprolactinemia in patients with schizophrenia (18). They suggested that the gene that regulates extrapituitary PRL production plays a role in regulating stress response by affecting the immune system. Moreover, prolactin may have an indirect role by affecting the hypothalamic-pituitary-adrenal axis. Within the brain, PRL acts as a neuropeptide to promote physiological responses related to reproduction, stress adaptation, neurogenesis, and neuroprotection (24). The action of PRL on the nervous system contributes to the wide array of changes that occur in the female brain during pregnancy and result in the attenuation of the hypothalamic-pituitary-adrenal axis (24). PRL moreover regulates neurogenesis both in the subventricular zone and in the hippocampus (24), which may mediate neuroplastic alterations during chronic stress. Therefore, alterations in the PRL system due to stress or exposure to substances that reduce neurogenesis or other conditions could contribute to mood and anxiety disorders. As far as we know, a possible association between *PRL* polymorphisms and depressive disorder had not yet been studied.

The aim of the present study was to investigate the possible relationship between being depressed and *BDNF* and *PRL* genotypes, on one hand, serum BDNF and PRL levels on the other, with special reference to severity of depression.

## Materials and Methods

### Patients

This study was carried out in accordance with the Code of Ethics of the World Medical Association (Declaration of Helsinki 1975, revised in Fortaleza, Brazil, 2013), and approved by the Institutional Medical Review Board of the Mental Health Research Institute (MHRI), Tomsk National Research Medical Center (protocol 49 from 23.04.12). Participants providing written informed consent were recruited from newly admitted patients to our Mental Health Research Institute.

Participants, aged 18–70 years old, were included based on a clinical diagnosis of depressive episode of a single (ICD: F32) or recurrent depressive disorder (ICD: F33) (25) with major depressive episode according to Mini International Neuropsychiatric Interview (M.I.N.I., 5.0.0) of clinically at least moderate severity (26). Exclusion criteria were non-Caucasian ethnicity; schizophrenia, decompensated personality disorders, pregnancy, or any relevant gynecological or endocrine (thyroid) disorder; relevant pharmacological withdrawal symptoms; or organic brain disorders (e.g., epilepsy, Parkinson's disease), treatment with antidopaminergic drugs (antipsychotic or antiemetic drugs), and treatment with antidepressant drugs during the preceding 6 months before admission. These exclusion criteria intended to avoid artificial intermingling of the genetic composition of the depressed patient group (ethnicity, other diagnosis) and/or to avoid factors which might influence the expression of PRL and/or BDNF (organicity, pregnancy, endocrinology, drugs).

### Control Group

The control group consisted of healthy volunteers who were actively recruited from society (mainly by advertisements spread within Tomsk universities and MHRI) and these individuals were also serving as controls for other patient populations investigated in the context of this project suffering from Parkinson's disease or schizophrenia. They were mentally and somatically healthy individuals of both genders and aged from 18 to 60 years, who provided written informed consent and were of White physical appearance. Potential control subjects were excluded if they presented chronic physical pathology in exacerbation, pregnancy, or any relevant gynecological or endocrine (thyroid) disorder; relevant pharmacological withdrawal symptoms; or organic brain disorders (e.g., epilepsy, Parkinson's disease) or mental disorders.

### Study Design and Ratings

After admission and obtaining informed consent, patients were diagnosed and assessed within 2 full days and treatment was initiated immediately thereafter. Depressed subjects were assessed with the 17-item Hamilton Depression Rating Scale (HAMD-17) (27, 28) and the Clinical Global Impression scale, severity (CGI-S) (29).

### Blood Sampling

Venous blood samples were drawn from median antecubital vein after an 8-h overnight fast and collected into evacuated tubes containing EDTA, for DNA extraction, or into tubes with a clot activator (CAT) to isolate the serum (BD Vacutainer). Blood samples with EDTA were stored in several aliquots at −20°C, until the DNA was isolated. Blood samples with CAT were centrifuged for 20 min at 2,000 g at 4°C to separate the serum; the serum was stored at −80°C, until prolactin or BDNF analysis.

### Multiplex Analysis

BDNF and prolactin concentration was measured in subject's serum by MAGPIX multiplex analyzer (Luminex, USA) using MILLIPLEX^®^ MAP kit (Merck, Germany).

### Genotyping

The DNA was genotyped for the studied genes in the Laboratory of Genetics of the University of Groningen with the MassARRAY^®^ System (Agena Bioscience™) and in the Laboratory of Molecular Genetics and Biochemistry of the Mental Health Research Institute using TaqMan^®^ SNP Genotyping Assays with “StepOnePlus” (Applied Biosystems). Rs1341239 (minor allele frequency; MAF: 0.38) was measured within the *prolactin* gene (position chr6:22303975), and rs6265 (MAF: 0.15), rs7124442 (MAF: 0.27), and rs11030104 (MAF: 0.23) were measured within the *BDNF* gene (position chr11:27658369; chr11:27655494; chr11:27662970, accordingly). Rs11030104 violated the Hardy-Weinberg equilibrium distribution and was excluded from further analyses. Given that rs11030104 was in high linkage disequilibrium (LD) with rs6265 (R-squared: 0.60) little information was lost by omitting rs11030104; the remaining SNPs were in very low LD to each other (see **Supplementary Image 1**).

### Statistical Analysis

Subjects with any missing genetic variant were removed and the remaining persons were combined in a single data set (**Supplementary Table 1**). Genetic associations were determined by sequentially regressing prolactin, BDNF, HAMD-17 (both as interval and as a high/low using the median 24 as cut-off), CGI-S, and depression (absent/present) on the available SNPs. Generalized linear model was used with either a Gaussian distribution (continuous or interval outcome data), or a binomial distribution (binary outcome data), and their respective canonical link functions. Genetic associations were evaluated assuming an additive model, this additivity assumption was evaluated using likelihood ratios tests providing no reasons to prefer a non-additive model. To reduce the influence of high prolactin and BDNF values these variables were analyzed on the natural logarithmic (ln) scale. Results are presented as mean difference (MD) or odds ratios (ORs) with 95% confidence intervals (95%CI).

## Results

Our total genotyped study population comprised of 186 depressed patients (17 male and 169 female) and 94 healthy persons (27 male and 67 female). Details are provided in **Table 1**. After excluding subjects without genetic information on all three study SNPs 217 remained of whom 138 suffered from depression, 41 subjects were male, and the mean age was 43.29 years (standard deviation: 13.58).

**Table 1:** Characteristics of genotyped individuals.

Healthy controls			
**Characteristics**	**n = 94**		
Gender	Male: 27 (28%); Female: 67 (72%)		
Mean age in years (sd)	33.82 (11.50)		
Median prolactin in ln(pg/ml) (Q1; Q3)	4.35 (2.51; 5.63)	6.14 (3.53; 9.23)	
Median BDNF in ln(pg/ml) (Q1; Q3)	4.23 (2.94; 6.51)		

**Patients with depression**			
**Characteristics**	**n = 186**		
Gender	Male: 17 (9%); Female: 169 (91%)		
Mean age in years (sd)	49.93 (10.75)		
Median HAMD-17 (Q1; Q3)	24 (21; 28)		
Median prolactin in ln(pg/ml) (Q1; Q3)	5.58 (3.53; 8.12)		5.46 (3.15; 10.37)
Median BDNF in ln(pg/ml) (Q1; Q3)	4.31 (3.10; 6.10)		

Nb sd, standard deviation; Q1 and Q3, quartile's 1 and 3; ln, natural logarithm.

**Figure 1:**
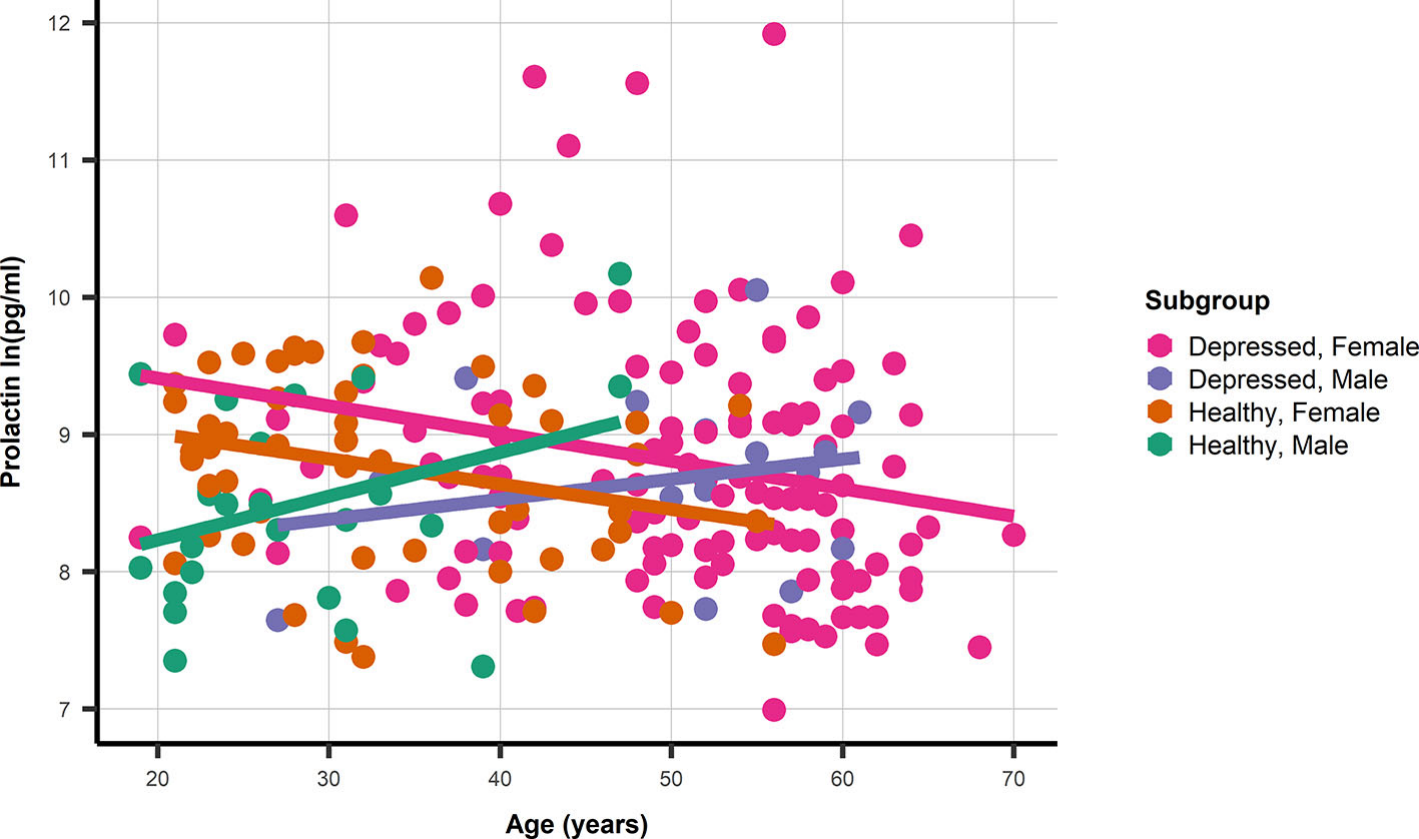
A dot and line plot of age and prolactin stratified by gender and depression status. N.b. Depicted lines are derived from subgroup specific linear regression models.

The association between age and prolactin was modified by gender, but not by depression status (**Figure 1**). With non-significant age association for male depressed: 0.01 ln(pg/ml) (95%CI −0.018; 0.047) and healthy men: 0.03 ln(pg/ml) (95%CI −0.003; 0.067), and significant associations between age and prolactin in depressed women: −0.02 ln(pg/ml) (95%CI −0.035; −0.005) as well as healthy women: −0.018 ln(pg/ml) (95%CI −0.035; −0.005). The *prolactin* SNP rs1341239 was not associated with prolactin levels −0.017 ln(pg/ml) (95%CI −0.182; 0.148).

**Figure 2:**
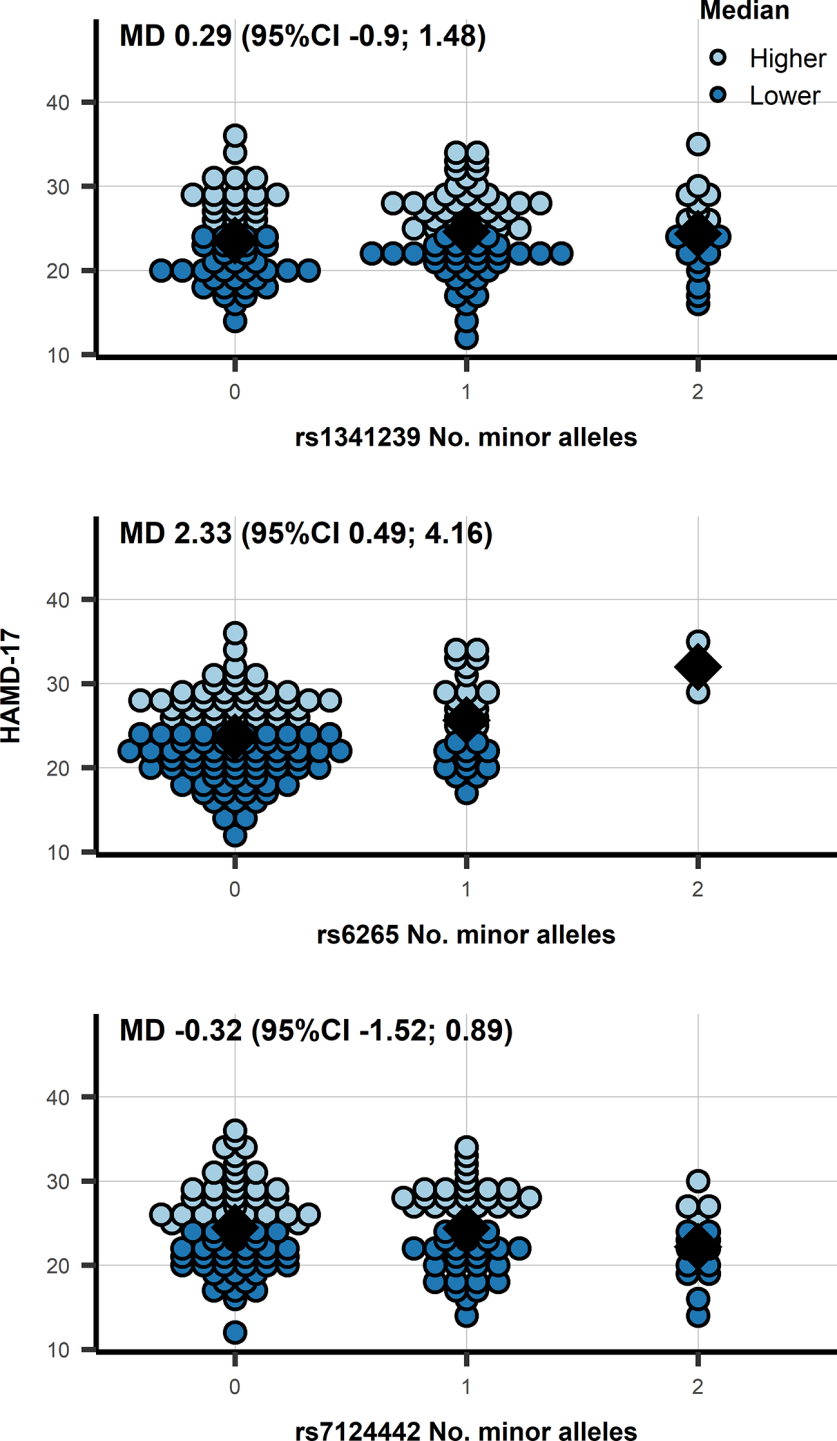
A dot plot of the HAMD-17 by number of minor allele in depressed patients, observations were color-coded based on a 24 cut-off. N.b. the diamond shape indicates the median HAMD-17; MD, mean difference from an additive genetic model; 95%CI, 95% confidence interval.

Similarly, the mean differences of *BDNF* SNPs did not show an association with BDNF: rs6265 −0.042 ln(pg/ml) (95%CI −0.198; 0.113), and rs7124442 0.006 ln(pg/ml) (95%CI −0.117; 0.130). Within depressed patients we observed an association of rs6265 with HAMD-17 (see **Figure 2**): mean difference (MD) 2.33 (95%CI 0.49; 4.16; *p* = 0.014). Categorizing HAMD-17 into high and low groups did not result in a similar significant effect for rs6265 (likely due to the limited number of depressed subjects with two minor alleles) (**Figure 2**), and neither did the odds ratio (OR) for depressed versus healthy: 0.62 (95%CI 0.35; 1.11; *p* = 0.110). The association with CGI-S mirrored those of HAMD-17 with significant findings for rs6265: MD 0.38 (95%CI 0.09; 0.66; *p* = 0.011), and without significant associations for rs1341239 MD −0.05 (95%CI −0.24; 0.13) and rs7124442 MD −0.08 (95%CI −0.26; 0.11).

The associations of prolactin and BDNF levels with the presence of depression was non-significant: OR 1.19 (95%CI 0.85; 1.68) for ln(prolactin), and OR 1.03 (95%CI 0.58; 1.84) for ln(BDNF). The genetic associations with depression status were similarly non-significant: rs1341239 OR 0.90 (95%CI 0.60; 1.36), rs6265 OR 0.62 (95%CI 0.35; 1.11), and OR 1.24 rs7124442 (95%CI 0.78; 1.97).

As mentioned before we did not observe any deviation for an additive genetic model. Furthermore, and despite the gender by age interaction on prolactin, we did not observe a similar gender or age modification of the genetic association with either depression status or prolactin/BDNF.

## Discussion

In our study, we found an association of rs6265 with the severity of depression according to the patient's HAMD-17 as well as CGI-S scores at admission to the hospital. The other studied genetic variants showed no significant relationship with the severity of depression upon hospital admission. Genetic associations were determined by sequentially regressing outcome variables on the available SNPs, which decreases the vulnerability for incorrect classification of disease in individuals; patients may suffer from different neurobiological disorders causing their major depression, and healthy probands may develop a major depression during their later life. We removed persons with missing genotype identity; this decreases the statistical power, but more importantly eliminates the distortion by studying different groups of persons for every SNP. We have no reason to believe that genotype data are missing in a non-random manner. We have also genotyped *BDNF* rs11030104, but unfortunately the results violated the Hardy-Weinberg equilibrium distribution and had to be excluded from further analyses. However, *BDNF* rs11030104 is partially in equilibrium with *BDNF* rs6265 (**Supplementary Image 1**), which limits the loss of these results. Neither the *PRL* SNP nor the *BDNF* SNPs were associated with the absolute levels of these neurotrophic factors. These actual PRL and BDNF levels were additionally not significantly associated with the presence of depression. This lack of association indicates that rs6265 is related to a pathogenetic element contributing to the vulnerability of becoming severely depressed and less to mediating the actual severity of this depression (by modulating actual BDNF expression). We expected that the likelihood of a possible association with a relevant genotype would be higher in severely depressed patients, but we could not demonstrate this due to the low number of patients with two minor alleles.

The composition of the studied patient population differs from the healthy control group. Certain selection bias is inevitable. Some healthy individuals will be prone to develop a depression and will probably get a depression during their later life. Individuals of female gender have a larger chance of becoming depressed, and also an older age could be an expected finding in at least moderate depressed patients (30). This is particularly relevant for the analysis using PRL levels. Premenopausal women have significantly higher PRL levels than postmenopausal female persons, who have not significantly different levels in comparison to men (31–33). This might be related to modulation of PRL dynamics by estrogen levels (31). Compared to the patient group only a very limited number of female controls of our study were of higher age. This might have decreased the difference in the mean PRL levels of depressed vs healthy persons. However, a similar relationship between PRL levels and age was obvious, and PRL levels were generally higher in depressed women in spite of the expected premenopausal versus postmenopausal study group composition (**Figure 1**). Nevertheless, it could be useful in the future to measure PRL well before midcycle in still menstruating women, check for (natural or exogenous) estrogen status in female persons, and match control subjects for age with women.

BDNF has been widely investigated with respect to major depressive disorder as well as bipolar disorder (4, 34). In major depression, there exists a clear relationship between BDNF levels and the depressive state, as well as the success of antidepressant therapy (4). The results of one of the last meta-analysis present evidence that supported the hypothesis that BDNF Val66Met polymorphism moderated the relationship between stress and depression, despite the fact that many included individual studies did not show this effect (35). We did not reproduce this finding concerning BDNF levels but observed an association between *BDNF* rs6265 and the severity of depression. The association observed in the present study could correspond to such a neuroplastic mechanism, which would make the individual becoming more easily and more severely depressed than other persons.

### Strength and Limitations

We studied 185 newly admitted patients who had not been treated with antidepressant drugs during their current depressive episode (operationalized to at least 6 months in patients with recurrent depression). Of these patients, 54.5% had never been treated with antidepressant drugs during their entire life. About half of these patients were seriously depressed according to the criteria of Zimmerman et al. (36). We only considered persons who were genotyped for all three considered variants, which largely improves the reliability of the observed associations. However, although reasonably sized for a biomarker study, our sample size after selecting these persons is relatively small for a pharmacogenetics study. Moreover, a certain gender imbalance between patients and controls is evident and postmenopausal patients were relatively scarce within the control group. Nevertheless, we observed a significant association between *BDNF* rs6265 and the severity of depression, which might mean that this reflects a relatively robust effect.

### Conclusions

We observed a significant association between *BDNF* gene variant rs6265 and the severity of depression in newly admitted, antidepressant treatment-free, depressed patients. Actual PRL and BDNF levels were not elevated sufficiently in depressed patients to reach statistical significance and were not associated with the studied genotypes.

## Data Availability Statement

Data are available from SI (ivanovaniipz@gmail.com) on reasonable request and with permission of MHRI.

## Ethics Statement

This study was carried out in accordance with the Code of Ethics of the World Medical Association (Declaration of Helsinki 1975, revised in Fortaleza, Brazil, 2013), and approved by the Institutional Medical Review Board of the Mental Health Research Institute (protocol 49 from 23.04.12). Participants providing written informed consent were recruited from psychiatric departments of the Mental Health Research Institute, Tomsk National Research Medical Center.

## Author Contributions

IL, FB, AL, and SI instigated and designed the study. AL and SI coordinated and supervised the study. NM, EH, and AS designed and performed the statistical analysis and contributed to writing the paper. SI wrote the study protocol and selected the SNPs. IL and AB monitored the study. GS collected clinical data. IL, LL, NV, and AB isolated DNA, genotyped the samples, and recorded all data in an Excel database. AB measured BDNF and PRL levels by Multiplex analysis. NB supervised the clinical work. SI, AL, and BW supervised the technical work. Ivan V. Pozhidaev and Diana Z. Paderina only genotyped the DNA samples within the laboratory of genetics of the University of Groningen and agree not to be co-authors. AL wrote the manuscript. IL, FB, BW, AS, and SI adapted the manuscript. All authors read the paper and agree with its content. Their role justifies their (co)-authorship to this paper.

## Funding

The research project was supported by RFBR grant №17-29-02205 «Development of a molecular-genetic panel of depressive disorders based on polymorphisms of the genes of neuronal kinases, neurotrophic proteins and genes of the serotonergic system» and by RFBR grant №14-04-01157 «The search for biomarkers of depressive and mood disorders».

## Conflict of Interest

The authors declare that the research was conducted in the absence of any commercial or financial relationships that could be construed as a potential conflict of interest.
