# Supplementary figures and images for: Association Between BDNF Gene Variant Rs6265 and the Severity of Depression in Antidepressant Treatment-Free Depressed Patients
Source: Front Psychiatry. 2020 Feb 12;11:38. doi: 10.3389/fpsyt.2020.00038 (PMC7028755; doi:10.3389/fpsyt.2020.00038)

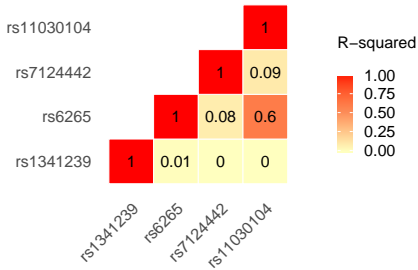

Supplement: Supplementary file 1 [file Image_1.pdf]
