# Supplementary material for: Association Between BDNF Gene Variant Rs6265 and the Severity of Depression in Antidepressant Treatment-Free Depressed Patients
Source: Front Psychiatry. 2020 Feb 12;11:38. doi: 10.3389/fpsyt.2020.00038 (PMC7028755; doi:10.3389/fpsyt.2020.00038)
Supplement: Supplementary file 2 [file Table_1.docx]

**Supplementary table 1 Characteristics of persons of analysed dataset**

| **Characteristics** | **Missing**  **observations** | **n = 217** |
| --- | --- | --- |
| Depression status | 0 | 138 (64%) |
| Male gender | 0 | 41 (19%) |
| Mean age in years (sd) | 0 | 43.29 (13.58) |
| Median HAMD-17 (Q1; Q3) | 79 | 24 (21; 28) |
| Median prolactin in ln(pg/ml) (Q1; Q3) | 3 | 8.16 (8.66; 9.25) |
| Median BDNF in ln(pg/ml) (Q1; Q3) | 34 | 8.02 (8.35; 8.72) |

Nb sd, standard deviation; Q1 and Q3, quartile’s 1 and 3; HAMD-17 was only recorded in depressed subjects; ln, natural logarithm.
